# Assessment and treatment of recurrent urinary tract infections in women: development of a questionnaire based on a qualitative study of patient expectations in secondary care

**DOI:** 10.1186/s12894-020-00764-6

**Published:** 2020-12-02

**Authors:** J. J. Pat, T. v. d. Aart, M. G. Steffens, L. P. W. Witte, M. H. Blanker

**Affiliations:** 1 Department of Urology, ISALA Clinics, Dokter van Heesweg 2, 8025 AB Zwolle, The Netherlands; 2 Department of General Practice, University of Groningen, University Medical Centre, Groningen, Hanzeplein 1, 9713 GZ Groningen, The Netherlands

**Keywords:** Female, Urinary tract infections, Questionnaire, Interview

## Abstract

**Background:**

To develop a questionnaire to facilitate the inventorying of women’s expectations for the assessment and treatment of recurrent urinary tract infection (UTI) in secondary care.

**Methods:**

Semi-structured interviews were conducted among women with recurrent UTI referred to our urology department. The interviews were conducted by one interviewer, recorded, transcribed verbatim, and analyzed thematically by two researchers. We first developed 35 questions to identify potential themes, and we then tested them among women with and without recurrent UTI. Changes were made according to the feedback received.

**Results:**

Six interviews were conducted before saturation was reached. Thematic analysis identified three themes: patient pathway, personal knowledge, and social implications. All respondents had received multiple antibiotic courses but no prophylactic antibiotic therapy, and although all were aware of some preventive measures, they wanted more information about their disease. However, some women were afraid to access information for fear of what they might learn. Recurrent UTI also significantly affected the daily lives all respondents. Some women expressed fears over frequent antibiotic use, and others felt that there must be something wrong with their body to have so many UTIs. Women expected the urologist to provide an explanation and to start adequate therapy for their recurrent UTI. We created a 32-item questionnaire based on these themes

**Conclusion:**

This study not only developed a questionnaire for use when assessing patient expectations of recurrent UTI management in secondary care but also provided novel insights into the thoughts, opinions, and expectations of women who are referred.

## Background

Urinary tract infections (UTIs) are among the most common bacterial infections worldwide [1], with estimates indicating that over 30% of all women will experience at least one UTI in their lives. Studies have shown that 20–50% of these women will experience at least one recurrence, defined as at least two UTIs in 6 months or at least three UTIs in 12 months [2, 3].

Several guidelines are available for the assessment and treatment of recurrent UTI, but there is no agreement on the optimal diagnostic process [1, 4, 5]. The Dutch Association of Urology commissioned a specific guideline about bacterial UTI in which it advised using a voiding diary, urine sediment assessment, and uroflowmetry in all patients with recurrent infection [1]. It only advocated additional diagnostic procedures, such as ultrasound or cystoscopy, in the presence of macroscopic hematuria or loin pain. The American Urology Association (AUA), Canadian Urology Association (CUA), and Society of Urodynamics, Female Pelvic Medicine and Urogenital Reconstruction (SUFU) recently published a guideline on recurrent UTI in which it was stated that cystoscopy and imaging should not be performed routinely [6]. Instead, assessments of women with recurrent UTI should focus on modifiable behaviors that are known to reduce the risk of recurrence, while excluding structural or functional abnormalities of the genitourinary tract.

Each year about 250 patients with recurrent UTI are referred to our outpatient clinic, and of these, approximately 20% have previously attended for this reason. It has been shown that patients often attend consultations in primary care with certain expectations for the outcomes [7]. However, these expectations have not been inventoried in secondary care for patients with recurrent UTI. Failure to consider these expectations is likely to affect consultation outcomes adversely. Using a questionnaire would help caregivers start important clinical conversations.

In this study, we aimed to assess patient expectations regarding the assessment and treatment of recurrent UTI in secondary care and to use the newly acquired information to develop a questionnaire on this topic.

## Methods

We conducted a qualitative study based on semi-structured interviews of women referred to our urology department with recurrent UTI. The interview guide was first tested with two patients. Women were excluded from participation if at least one of the following criteria was met: referral for complicated UTI, known urological malignancy, intermittent or continuous catheterization, pregnancy, or insufficient proficiency with the Dutch language. Eligible patients were given an invitation letter and a written informed consent form. The study was approved by our local medical ethics committee under approval number 180319.

After patients signed the informed consent form, a single researcher held face-to-face or telephone interviews according to an interview guide (Additional file 1). To minimize interviewer bias, the researcher (TvdA) was a trained medical student who clarified to participants that she was not a medical doctor. All interviews were recorded and transcribed verbatim. The interviewer started with an open question about the patient’s expectations of the visit to the urologist, and although open questions were used throughout the interview, the interviewer was free to seek elaboration on initial responses. Additional interviews were held until saturation was reached, which was defined as obtaining no new information from two consecutive interviews. The questionnaire was written in Dutch and translated to English by a native speaker. The English version was then translated back to Dutch to check if the English version matched the original questionnaire.

### Analysis

We performed thematic analysis using Atlas.TI 8® (ATLAS.ti Scientific Software Development GmbH). Two researchers (TvdA and JJP) separately read and re-read the transcripts to become familiar with the data, then analyzed the text and added codes to the responses. Open coding was used, allowing the codes to be developed and adjusted as the interview transcripts were analyzed. After the initial coding, we evaluated the codes and organized them into themes, re-read the interviews with the themes in mind, and formulated questions that covered the content of those themes. We took care to ensure that the questionnaire was suitable for patients with low literacy. No analysis of internal consistency was performed because the questionnaire was intended to generate an inventory of opinions rather than to categorize the outcomes. Finally, the questionnaire was completed by a diverse group of women with and without recurrent UTI to assess its readability. Any resulting changes were made according to the feedback received.

## Results

Saturation was reached after six interviews (four face-to-face and two by telephone) lasting 35–60 min each. The women were aged 32–86 years and all had suffered recurrent UTI for at least 1 year. The presenting symptoms consisted of dysuria, abdominal pain, frequency, urinary incontinence, and occasional fever. Analysis of the interviews yielded three main themes: medical route, knowledge of the condition, and social and psychological impact. The following sections describe each theme in turn.

### Medical route

All respondents described that their general practitioner (GP) had tested their urine for a UTI when they had symptoms of a UTI and prescribed antibiotics when positive. They reported having received antibiotic treatment more than once for UTI, but also noted that some antibiotics worked better than others. Some respondents reported a delay in antibiotic treatment because they had to wait for the results of the urine culture before a GP was willing to start antibiotic therapy.

Well then, that is what you do at the GP. You bring in your urine and after a day you’re supposed to make a call. It always makes me feel like: oh, time to call again.

Some respondents had undergone physical examination, and only one reported completing a voiding diary.

Interviewer: *What tests were done by the GP?*

Respondent: *Well actually, well yes, not internally. He has occasionally patted me on the back asking if I felt pain here or there.*

None of the respondents had used antibiotics prophylactically, although one patient was aware that this was a therapeutic option.

Well, I thought that he maybe, that you maybe had–for example–a standard antibiotic treatment, because I’ve heard that you could preventively take something like two antibiotic pills.

All respondents were given advice about prophylaxis by their GPs, such as taking cranberry tablets, ensuring adequate fluid intake, and performing post-coital voiding.

And I’ve had it prescribed by the GP. But nowadays I just get them at my local pharmacy, those Cranberry tablets, because they are a lot cheaper there; but yes, I do make use of them.

Some respondents described repeatedly asking their GP for a urology referral, but having to wait up to 2 months for an appointment after the referral was made.

Yes, yes, I did visit there, but of course I insisted on it.

One respondent stated that her GP advised that a urology referral was needed and that she agreed because she felt like there had to be something wrong to get so many UTIs.

And then I told them my story and that I’ve had it all so and so. And then they said, “well it’s about time that you visit a urologist.” And then I responded, “yes, I happen to agree, now that I’ve had it so often in succession and it still isn’t gone, there is probably something not completely in order indeed.”

Full recovery was not always the main goal of patients. Some described that they would be satisfied with a reduction in the frequency of UTIs.

Interviewer: And when would you be satisfied?

Respondent: Well yeah, if I notice any results.

Interviewer: So, if you only had cystitis once a year, would you say the treatment was successful?

Respondent: Yes.

Another described that they would be satisfied with a clarification about their UTI.

Interviewer: And when would you be satisfied?

Respondent: Well at first instance I would say, with regard to tomorrow's examination, that I would be happy if I just had more clarity about my condition.

### Patient knowledge of the condition

Respondents described a variety of measures they had adopted to prevent recurrence, including taking cranberry tablets and ensuring adequate fluid intake, post-coital voiding, and intimate hygiene. Some respondents were able to list all preventive measures, while others only named one or two. Most respondents had looked for information about recurrent UTI on the internet, but others did not want to because they feared what they would find.

No, no, no I totally can’t. Yes, I have such a tablet but no, I don’t look up anything. No, not at all. Oh, no I don’t, I really don’t want to know.

There were also differences in the information wanted by respondents. Some only wanted information about the causes, some only wanted information about the therapeutic options, and others wanted both. All respondents stated that they received information from the hospital about their appointment and were able to explain both the voiding diary they had to complete and the uroflowmetry to be performed. They felt like they had received enough information on these matters before the consultation.

Alright. And what exactly is this “pot” research? Well it goes, that’s exactly it, it measures how fast or how slowly I pee, how much leaves my bladder and what its composition is, so to speak.

Most respondents did not know about other potential diagnostic tests. One respondent was aware of cystoscopy because she had been referred previously.

He’ll probably have to look into the bladder anyway. Because that’s what happened the last time. And then you can really see that a bladder isn’t quite … clean from the inside either.

### Social and psychological impact

All respondents described that their daily lives were affected by the symptoms of recurrent UTI. They all had active social lives and some had needed to take measures in the event of urinary incontinence when they visited someone (such as taking sanitary napkins or clean underwear). The hospital visit was also a burden for some because they were unable to arrive with a full bladder because of symptoms. Others reported that they could not talk about their problems with relatives because they felt this was inappropriate.

And urology is of course still a subject that isn’t that openly discussed at parties and the like.

Another respondent felt guilty because she regularly handed her urine into the general practice and thought that she was complaining too much.

But sometimes I do get the feeling that you’re a bit of a whiner. That’s not how my GP responds, but I do feel like we, as patients, while we are confronted with the hindrance and pain and constant annoyance of having to pee so often, invoke a reaction of: “oh here comes another one with his pee.” Yeah then they shouldn’t have invented them in the first place, urologists.

Respondents described a variety of worries. The most common was about taking regular antibiotics, which many felt was harmful to their bodies.

Because I don’t want to take antibiotics every time since that isn’t good for your body either.

Another common worry was about the cause of the recurrent UTI. Respondents felt that there had to be something wrong with their bodies to be getting so many UTIs.

Why do you get a cystitis every time? There has to be something wrong somewhere.

### The questionnaire of patient expectations

We formulated a 32-item questionnaire based on the major themes identified in the participant interviews. The questions varied in style, including statements that required a yes or no answer, statements that required answers on 5-point Likert scales, open-end questions and questions that required multiple answers with room for free text responses. Next, we tested the questionnaire in interviews with five patients and five non-patients. This revealed that three questions needed to be withdrawn due to similarities with other questions and that ten questions needed to be rewritten to improve comprehensibility. The final questionnaire consisted of 32 questions and was entitled the ESC-rUTI questionnaire (Expectations of Treatment in Secondary Care–Recurrent UTI). A copy of the English translation is provided in Additional file 2.

## Discussion

This is the first study to explore patient expectations of secondary care assessment and treatment for recurrent UTI. Thematic analysis of interview data uncovered three themes: medical route, patient knowledge of the condition, and social and psychological impact.

### Medical route

The first theme concerned pathways taken by patients and broadly covered the trajectory from symptom onset to referral. The most notable finding was that none of the respondents received prophylactic antibiotics from their GP, despite this being a clear recommendation in the Dutch General Practitioner guideline [8]. We plan to investigate this further in a future questionnaire-based study among GPs.

Some respondents reported that they had frequently asked their GP for a urology referral, indicating that referral is not always deemed necessary by the GP. A prospective survey of all referrals over a 5-month period from generalists to four selected academic sub-specialisms in the United States concluded that one-fifth of referral decisions were influenced by patient requests [9]. Furthermore, we found indicators that not all patients expect to achieve complete cure.

### Patient knowledge of the condition

Although all respondents were able to name some preventive measures for their recurrent UTI, only some were able to mention all of them, indicating room for improvement in patient education. Most respondents wanted more information on either what they could do to prevent UTI, what causes UTI or both. Both these findings are supported by a recent qualitative study in the primary care setting regarding UTI [10]. Most respondents took the initiative to search for the information they needed, but some were afraid to do so because of what they would find. All respondents were able to explain the diagnostic procedures that would initially be performed in the hospital based on the information they had been sent, though only one was able to explain cystoscopy. Overall, there was a lack of knowledge among respondents, which indicated a need for reliable information about the diagnostic and treatment options for recurrent UTI.

### Social and psychological impact

All respondents in our study described that their social lives had been greatly affected by their UTIs. This finding is supported by a large web base study that showed that recurrent UTI have a significant impact on quality of life [11]. Some were concerned about the cause of their UTI because they felt that there had to be something wrong with them to have UTIs on such a regular basis. Others were worried that regularly taking antibiotics would harm their bodies. These worries have previously been described in a qualitative study regarding public beliefs about antibiotics, infection, and resistance [12].

### Strengths and limitations

The strengths of this study include the use of qualitative semi-structured interviews until saturation. The responses highlighted the important issues and expectations among patients concerning the management of recurrent UTI. However, this study also had several limitations. It was notable, for example, that the findings were based on data from a single center, meaning that we cannot be certain if they can be extrapolated to the general population. Although one respondent had already been seen in this clinic for recurrent UTI, her expectations were similar to those expressed by the other respondents Patient inclusion in our study might have introduced a certain response bias, as nearly all patients will report some level of bother. However, the level of bother may well differ between patients, and impact their expectations. Future research will elucidate this. Although the results of open-end questions are more difficult to interpret we chose to use them in order to test knowledge and not recognition. It also helps to gain knowledge in misconceptions.

Though this questionnaire will not change patient expectations, we think that knowledge of such expectations, may be helpful to adjust assessment and treatments to individual needs. Gaining insight in the expectations of the group of patient helps us align general patient information with this.

### The final questionnaire

Despite these limitations, we were able to create the 32-item ESC-rUTI questionnaire. We anticipated that this will prove useful in providing information about a given patient’s trajectory from symptom onset to referral, knowledge of the disease, concerns about the social and psychologic impact of the disease, and expectations for the hospital visit. However, we opted not to perform test–retest analyses because we do not plan to use the questionnaire to measure treatment effects. Instead, the questionnaire will only be used to create an inventory of expectations before an outpatient visit; thus, we believe that additional validation is not necessary. The final questionnaire covers a variety of relevant subjects, including the impact of symptoms on daily life, satisfaction with the GP, peer support, and expectations regarding diagnostics, education, treatment, and recurrent UTI (Additional file 2).

As the questionnaire was developed to be used in the Netherlands, the English version was not tested for readability with native English speakers. This needs to be done before using the English version.

## Conclusion

This study provides insights into the expectations of newly referred patients with recurrent UTI. Follow-up research using the developed questionnaire has already been started to quantify these expectations and to see how they affect the care we provide.

## Supplementary Information

**Additional file 1.** Interview guide patient expectations regarding diagnosis and treatment of referral for recurrent urinary tract infections.

**Additional file 2.** English translation of the ESC-rUTI questionnaire.

## Data Availability

The datasets used or analysed during the current study are available from the corresponding author on reasonable request.

## Abbreviations

UTI: Urinary tract infection
AUA: American Urology Association
CUA: Canadian Urology Association
SUFU: Society of Urodynamics, Female Pelvic Medicine and Urogenital Reconstruction
GP: General Practitioner
ESC-rUTI: Expectations of Treatment in Secondary Care–Recurrent UTI
