# Supplementary material for: Assessment and treatment of recurrent urinary tract infections in women: development of a questionnaire based on a qualitative study of patient expectations in secondary care
Source: BMC Urol. 2020 Dec 2;20:190. doi: 10.1186/s12894-020-00764-6 (PMC7709265; doi:10.1186/s12894-020-00764-6)
Supplement: Supplementary file 1 — Additional file 1. Interview guide patient expectations regarding diagnosis and treatment of referral for recurrent urinary tract infections. [file 12894_2020_764_MOESM1_ESM.docx]

**Interview guide patient expectations regarding diagnosis and treatment of referral for recurrent urinary tract infections**

**Introduction**

Welcome, we thank you for participating in this study. I am a researcher within this study. I’m not a medical specialist, I won’t advice or prescribe any form of treatment. Anything we discuss in this interview is anonymous, this interview will not be shared with your doctor.

Do you have any questions before we start with the interview?

For our study it is important to review the interview by listening it back, so we will record the interview. We won’t record names, any names mentioned in this interview will be deleted later on. Do you agree we will record this interview? I will start recording now.

This is interview number…

**Interview**

What do you expect from this referral? What do you expect from your urologist?

Can you briefly describe for how long you’ve had symptoms?

Can you briefly describe your symptoms?

Do you have any symptoms at the moment?

Where your symptoms the reason for referral?

What did your GP tell you about recurrent urinary tract infections?

What did your GP tell you about the referral?

What information did you receive from the hospital regarding your referral?

Did you understand the information?

Do you know the possible outcomes of these diagnostic tests?

Did you personally look up information about recurrent urinary tract infections?

Why / why not?

What did you find?

Did you receive information from friends or family?

What do you expect to be the outcome of the diagnostic tests?

What treatment options do you know for recurrent urinary tract infections?

What treatment do you think the urologist will prescribe?

Which result do you want to achieve? When are you satisfied?
