# Supplementary material for: Assessment and treatment of recurrent urinary tract infections in women: development of a questionnaire based on a qualitative study of patient expectations in secondary care
Source: BMC Urol. 2020 Dec 2;20:190. doi: 10.1186/s12894-020-00764-6 (PMC7709265; doi:10.1186/s12894-020-00764-6)
Supplement: Supplementary file 2 — Additional file 2. English translation of the ESC-rUTI questionnaire. [file 12894_2020_764_MOESM2_ESM.docx]

This questionnaire concerns recurrent-cystitis. You have been referred to the hospital for this. The questionnaire will start with a few questions regarding your symptoms. After this, we will ask you about the policy of your GP. We will also ask you about your own thoughts and worries related to the symptoms you are experiencing. Finally, we will ask you for your expectations towards the urologist. There are no wrong answers. What counts, is what is important for you. For this reason, please respond with an answer that best matches your situation. Try to answer all questions. Thank you very much for your effort!

In case you wish to answer this questionnaire online, go to the following link or scan the QR code. The password is: xxx

1: What is your respondents code? (You will find this on the consent form)

2: If I suffer from symptoms, my GP (or their assistant) checks my urine in their practice.

Always Usually Half of the time Sometimes Never

3: The GP sends my urine to the hospital for examination

Never Once More than once I do not know

4: The GP physically examined me (back/belly, genital organ).

Yes No

5: The GP made me keep a voiding-list (also known as a voiding-diary).

Yes No

6: The GP treated my cystitis with antibiotics.

Always Usually Half of the time Sometimes Never

7: The GP gave me a treatment (estrogens or maintenance treatment with antibiotics) to prevent cystitis. In case you answer this question with “no”, continue to question 9.

Yes No

8: Which treatment did the GP give you to prevent cystitis? (Multiple answers possible).

Antibiotics Estrogens (cream or vaginal capsule)

Other:

9: I know what I can do myself to prevent cystitis. In case you answer this question with “no”, continue to question 12.

Yes No

10: What can you do yourself to prevent cystitis?

Free text

11: Did you put this to use?

Yes No

12: I would like to receive more information about recurrent cystitis. In case you answer this question with a “no”, continue to question 14.

Yes No

13: I would like to look up more information regarding recurrent cystitis myself.

Yes No

14: I am afraid / I worry about what I will find, if I look up more information about recurrent-cystitis.

Completely agree Agree Neutral Disagree Completely disagree

15: I would like to receive more information about the causes of cystitis.

Yes No

16: I would like to receive more information about the treatment of cystitis.

Yes No

17: I find it easy to discuss my symptoms with others. In case you answer this question with “(completely) agree”/”neutral”, continue to question 19.

Completely agree Agree Neutral Disagree Completely disagree

18: I (completely) disagree because… (multiple answers possible)

I have the feeling that there is a taboo on the subject of urology

I am ashamed of my symptoms

Other:

19: The symptoms affect my daily life

Completely agree Agree Neutral Disagree Completely disagree

20: I think my GP understands me.

Completely agree Agree Neutral Disagree Completely disagree

21: I feel understood / supported by my environment.

Completely agree Agree Neutral Disagree Completely disagree

22: Visiting the hospital is a big burden to me.

Completely agree Agree Neutral Disagree Completely disagree

23: I worry about the cause of my recurrent-cystitis.

Completely agree Agree Neutral Disagree Completely disagree

24: There has to be a cause of my recurrent-cystitis.

Completely agree Agree Neutral Disagree Completely disagree

25: I have an idea about the possible cause of my symptoms.

Completely agree Agree Neutral Disagree Completely disagree

26: I worry about having to use antibiotics all of the time.

Completely agree Agree Neutral Disagree Completely disagree

27: My GP has taken the initiative to refer me.

Completely agree Agree Neutral Disagree Completely disagree

28: I have had to insist to my GP in order to be referred.

Completely agree Agree Neutral Disagree Completely disagree

29: I expect that the urologist can explain to me why I suffer from cystitis so often.

Completely agree Agree Neutral Disagree Completely disagree

30: I expect that the urologist is able to remedy my symptoms.

Completely agree Agree Neutral Disagree Completely disagree

31: I expect that the urologist will prescribe a different treatment than my GP does.

Completely agree Agree Neutral Disagree Completely disagree

32: I will be satisfied if I have an explanation for my complaints, even if this does not remedy my symptoms.

Completely agree Agree Neutral Disagree Completely disagree

33: I will be satisfied if no dysfunctions are found, even if this does not explain my symptoms and they do not disappear.

Completely agree Agree Neutral Disagree Completely disagree

This is the end of the questionnaire. Thank you again for your effort. You can return this questionnaire together with the consent form, in the added retour-envelope.
